# Diagnostic value of ultrasound in pediatric acute abdominal pain: a cross-sectional study from a tertiary center in Southern Iran

**DOI:** 10.1186/s12887-025-06059-9

**Published:** 2025-09-25

**Authors:** Mehdi Kavari, Robab Sadegh, Reza Golchin Vafa

**Affiliations:** 1 School of Medicine, Shiraz University of Medical Sciences, Shiraz, Iran; 2 Emergency Medicine Department, School of Medicine, Shiraz University of Medical Sciences, Shiraz, Iran

**Keywords:** Abdominal pain, Acute abdomen, Ultrasonography

## Abstract

**Background:**

Acute abdominal pain is a common yet diagnostically challenging presentation in pediatric emergency care due to nonspecific symptoms. While ultrasound offers a safe and accessible diagnostic tool, its clinical utility and correlation with laboratory markers remain understudied in many regions, including Iran. This study aimed to evaluate the prevalence and diagnostic value of ultrasound findings and their association with clinical and laboratory indicators.

**Methods:**

This cross-sectional study included 174 children aged 5–18 years who presented with acute abdominal pain to the emergency department of Namazi Hospital, Shiraz, Iran, between 2021 and 2022. Demographic data, clinical features, laboratory results, and ultrasound findings were recorded. Associations between sonographic abnormalities and clinical/laboratory parameters were analyzed using appropriate statistical tests.

**Results:**

Ultrasound revealed abnormal findings in 59 (33.9%) patients. The most common sonographic diagnoses were abdominal free fluid (7.5%), appendicitis (4.6%), renal stones (2.9%), and intestinal obstruction (2.9%). Right lower quadrant pain and elevated CRP levels (> 20 mg/L) were significantly associated with appendicitis (*p* = 0.018 and *p* = 0.012, respectively). Positive urine protein and blood were significantly associated with renal stones (*p* = 0.01 and *p* = 0.003). The majority (79.3%) of ultrasounds were normal, highlighting the role of clinical assessment in determining the necessity of imaging.

**Conclusions:**

Ultrasound is a valuable diagnostic tool in pediatric patients with acute abdominal pain, identifying both common and rare conditions. The study highlights the importance of clinical assessment in determining the need for further imaging and the necessity of localized research to inform diagnostic protocols in regional healthcare settings.

## Introduction

Acute abdominal pain is a frequent and challenging symptom encountered in pediatric emergency departments globally. It contributes significantly to emergency department visits, spanning from mild, self-resolving conditions to critical, life-threatening surgical emergencies [1, 2]. In children, Assessing abdominal pain can be especially challenging due to the frequently nonspecific nature of symptoms, limited verbal communication abilities in younger children, and a broad spectrum of differential diagnoses that differ across age groups [3]. Accurate and timely diagnosis is critical to avoid complications and unnecessary surgical interventions while ensuring appropriate management [4].

Ultrasound has emerged as a crucial diagnostic modality in assessing acute abdominal pain in pediatric patients [5]. As a non-invasive, readily available, and radiation-free modality, it is often preferred over other imaging techniques like computed tomography (CT) in the pediatric population [6]. Its utility spans a broad spectrum of conditions, including appendicitis, intussusception, mesenteric lymphadenitis, and ovarian or testicular torsion, among others [7]. The sensitivity and specificity of ultrasound in diagnosing appendicitis, for example, have been well-documented, making it a cornerstone of the diagnostic approach in many emergency settings [8].

Despite its widespread use, the prevalence of specific ultrasound findings in children with acute abdominal pain remains under-researched in many regions, particularly in developing countries [9]. Comprehending these patterns is essential for customizing diagnostic protocols, enhancing resource distribution, and improving patient outcomes [10].

Moreover, regional variations in disease prevalence, healthcare infrastructure, and patient demographics underscore the need for localized data to inform clinical practice [11]. However, there is a paucity of data regarding the sonographic findings in pediatric patients presenting with acute abdominal pain to its emergency department [12]. This gap highlights the need for a comprehensive evaluation to assess the prevalence of various sonographic findings, their diagnostic implications, and their contribution to clinical decision-making [13]. This study aims to provide a detailed assessment of sonographic findings in pediatric patients with acute abdominal pain presenting to the emergency department of Namazi Hospital in 2021 and 2022. Through the analysis of these findings, we aim to provide valuable insights into the diagnostic framework, promote evidence-based practices, and ultimately improve the quality of care for pediatric patients in this context.

## Methods

### Study design

This cross-sectional, retrospective study was conducted in the Emergency Department (ED) of Namazi Hospital, a tertiary care center in Shiraz, Iran. The hospital serves as a referral hub for pediatric emergencies in the region. The study analyzed data from pediatric patients presenting with acute abdominal pain between January 1, 2021, and December 31, 2022.

### Study population

The study included pediatric patients aged 5 to 18 years who presented to the ED with acute abdominal pain and underwent abdominal ultrasonography (AUS) as part of their diagnostic workup. Patients were excluded if their ultrasound findings were inconclusive or if they had incomplete medical records.

### Data collection

Patient data were extracted from the hospital’s electronic medical records and radiology database. Information collected included:

1. Demographic Data: Age, sex, and presenting symptoms.
2. Clinical Information: Duration of symptoms, associated clinical signs, and preliminary diagnoses.
3. Ultrasound Findings: Results of AUS, including identified abnormalities such as appendicitis, intussusception, bowel obstruction, mesenteric lymphadenitis, hernias, or any other pathologies.

### Ultrasonography protocol

All AUS examinations were performed using high-resolution ultrasound machines by experienced radiologists specialized in pediatric imaging. The standard protocol for AUS included:

- Imaging Techniques: Use of linear and curvilinear transducers, depending on the patient’s body habitus and clinical indication.
- Assessment Areas: Complete evaluation of the abdomen, including the appendix, intestines, lymph nodes, liver, spleen, kidneys, and bladder.
- Diagnostic Criteria: Standardized sonographic criteria were used to diagnose common conditions. For example
  - Appendicitis: Enlarged, non-compressible appendix > 6 mm in diameter with peri appendiceal echogenicity.
  - Intussusception: Presence of the “target sign” or “doughnut sign.”
  - Bowel Obstruction: Dilated bowel loops with fluid levels and increased peristalsis.
  - Mesenteric Lymphadenitis: Multiple enlarged mesenteric lymph nodes > 10 mm in short axis.

### Outcome measures

The primary outcome was the prevalence of specific sonographic findings among the study population. Secondary outcomes included the correlation between ultrasound findings and associated clinical symptoms.

### Data analysis

Data were analyzed using SPSS version 25 (IBM Corp., Armonk, NY, USA). Descriptive statistics were employed to summarize the characteristics of the study population. Continuous variables, such as age and duration of symptoms, were expressed as mean ± standard deviation (SD) for normally distributed data or as median with interquartile range (IQR) for non-normally distributed data. Categorical variables, including sex, ultrasound findings, and associated conditions, were presented as frequencies and percentages.

The normality of continuous variables was assessed using the Kolmogorov-Smirnov or Shapiro-Wilk tests. Depending on the results of these tests, appropriate statistical methods were applied. Independent samples t-tests were used to compare normally distributed continuous variables between groups, while the Mann-Whitney U test was applied for non-normally distributed data. For categorical variables, comparisons were made using chi-square tests or Fisher’s exact tests when expected frequencies were below five. Additionally, the correlation between ultrasound findings and clinical symptoms was evaluated using Pearson or Spearman correlation coefficients, depending on data distribution. A p-value of < 0.05 was considered statistically significant for all analyses.

### Ethical considerations

The study was approved by the Ethics Committee of Shiraz University of Medical Sciences (IR.SUMS.MED.REC.1403.534). As this was a retrospective study, informed consent was waived. All data were anonymized to protect patient confidentiality in accordance with the Declaration of Helsinki.

## Results

**Fig. 1:**
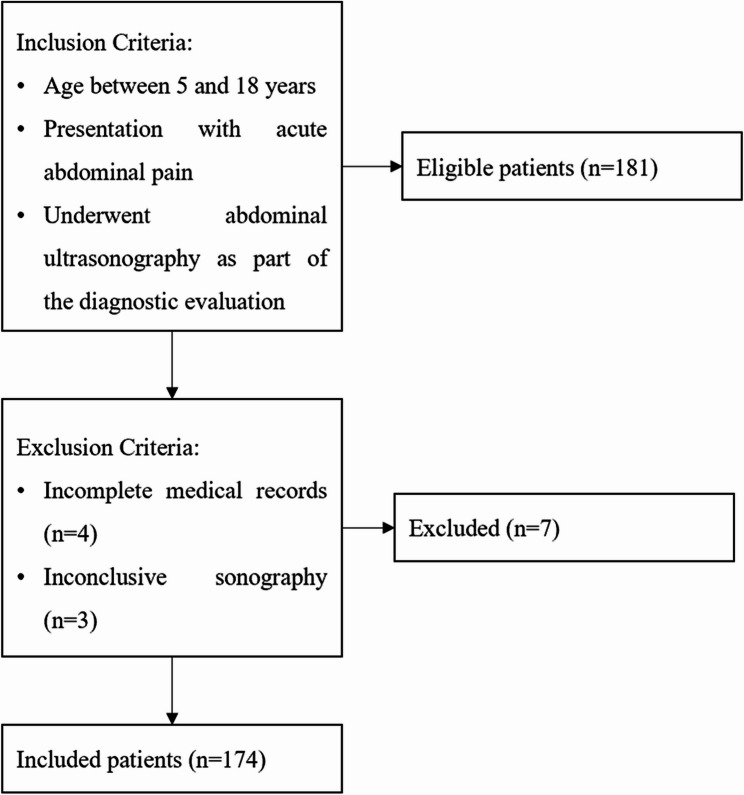
Flowchart of patient selection process detailing inclusion and exclusion criteria for pediatric patients presenting with acute abdominal pain

A total of 174 pediatric patients were included (Fig. 1), with a mean age of 6.5 ± 2.5 years. The study population comprised 91 males (52.3%) and 83 females (47.7%). Laboratory findings revealed a mean blood urea nitrogen (BUN) level of 12.6 ± 5.9 mg/dL and a mean creatinine (Cr) level of 0.61 ± 0.41 mg/dL. The mean white blood cell (WBC) count was 10,333.1 ± 3971.2/µL, and the mean hemoglobin (Hb) level was 11.9 ± 1.6 g/dL. The mean C-reactive protein (CRP) level was 27.4 ± 37.5 mg/L. Urinalysis findings showed that proteinuria was present in 6 patients (3.4%), hematuria in 13 patients (7.5%), and ketonuria in 10 patients (5.7%). Pyuria was detected in 7 patients (4%), while bacteriuria was observed in 6 patients (3.4%). These data provide an overview of the baseline clinical characteristics and laboratory profiles of the study population (Table 1).

**Table 1:** Baseline demographic and laboratory characteristics of the study population

		Participants (*n* = 174)
Age (Mean ± SD)		6.5 ± 2.5
Gender (n, %)	Female	83 (47.7)
	Male	91 (52.3)
BUN (Mean ± SD)		12.6 ± 5.9
Cr (Mean ± SD)		0.61 ± 0.41
WBC (Mean ± SD)		10333.1 ± 3971.2
Hb (Mean ± SD)		11.9 ± 1.6
CRP (Mean ± SD)		27.4 ± 37.5
Urine Protein (n, %)	Positive	6 (3.4)
Urine Blood (n, %)	Positive	13 (7.5)
Urine Ketone (n, %)	Positive	10 (5.7)
Urine WBC (n, %)	Positive	7 (4)
Urine Bacteria (n, %)	Positive	6 (3.4)

Among the 174 participants, the majority experienced generalized abdominal pain (66.7%, *n* = 116). Pain localized to the right lower quadrant (RLQ) was reported in 14.9% (*n* = 26), while periumbilical pain was observed in 10.3% (*n* = 18). Other less common pain locations included the left lower quadrant (LLQ) and hypogastric region, each reported in 2.9% (*n* = 5), and the epigastric region in 2.3% (*n* = 4). The most frequently reported associated symptom was nausea and vomiting, occurring in 40.2% of patients (*n* = 70), followed by fever in 26.4% (*n* = 46). Diarrhea and constipation were each observed in 10.3% of patients (*n* = 18). Additional symptoms included anorexia (8%, *n* = 14), tenderness (9.2%, *n* = 16), and abdominal distention (2.9%, *n* = 5). Less commonly, dysuria (1.1%, *n* = 2) and irritability (1.7%, *n* = 3) were reported. The duration of symptoms ranged widely, with a mean duration of 2.8 ± 2.3 days. These findings highlight the varied presentations of abdominal pain and associated clinical features in pediatric patients presenting to the emergency department (Table 2).

**Table 2:** Characteristics of abdominal pain and associated symptoms in the study population

		Participants (*n* = 174)
Abdominal Pain Location (n, %)	Generalized	116 (66.7)
	RLQ	26 (14.9)
	LLQ	5 (2.9)
	Periumblical	18 (10.3)
	Epigastric	4 (2.3)
	Hypogastric	5 (2.9)
Associated Sign and Symptoms (n, %)	Diarrhea	18 (10.3)
	Constipation	18 (10.3)
	Fever	46 (26.4)
	N/V	70 (40.2)
	Dysuria	2 (1.1)
	Irritability	3 (1.7)
	Anorexia	14 (8)
	Tenderness	16 (9.2)
	Distention	5 (2.9)
Duration (Mean ± SD)	Days	2.8 ± 2.3

Out of the total study population, 115 patients (66%) had normal sonographic findings. Among the abnormal findings, abdominal free fluid was the most prevalent, observed in 13 patients (7.5%). Appendicitis was identified in 8 patients (4.6%), while renal stones and intestinal obstruction were each detected in 5 patients (2.9%). Intussusception was noted in 4 patients (2.3%). Less common findings included mesenteric lymphadenitis and reactive lymph nodes, each seen in 3 patients (1.7%). Cholecystitis, hydronephrosis, pseudo-kidney appearance, and inguinal hernia were each reported in 2 patients (1.1%). Rare sonographic findings, each present in one patient (0.6%), included mesenteric inflammation, vesicoureteral reflux (VUR), right lower quadrant lymphadenopathy (RLQ LAP), urinary bladder distention, cervical lymphadenopathy, distended gallbladder with sludge, ileus, ovarian cyst, collection posterior to the urinary bladder, fatty liver, liver cyst, urinary bladder debris, intestinal infection, and kidney stasis. Although free fluid was the most commonly observed abnormal sonographic finding, none of the patients in this group were diagnosed with complicated appendicitis. These findings highlight the diversity of underlying pathologies in pediatric patients presenting with acute abdominal pain, emphasizing the diagnostic value of sonography in this clinical setting (Table 3).

**Table 3:** Sonographic findings in pediatric patients with acute abdominal pain

	*n*	% (of 174 total cases)	% (of 59 abnormal cases)
Abdominal Free Fluid (n, %)	13	7.5	22
Appendicitis (n, %)	8	4.6	13.5
Renal Stone (n, %)	5	2.9	8.4
Intestinal Obstruction (n, %)	5	2.9	8.4
Intussusception (n, %)	4	2.3	6.7
Mesenteric Lymphadenitis (n, %)	3	1.7	5
Reactive lymphnodes (n, %)	3	1.7	5
Cholecystitis (n, %)	2	1.1	3.3
Hydronephrosis (n, %)	2	1.1	3.3
Psoudo kidney (n, %)	2	1.1	3.3
Inguinal Hernia (n, %)	2	1.1	3.3
Acute Hepatitis (n, %)	1	0.6	1.6
Mesentric Inflammation (n, %)	1	0.6	1.6
VUR (n, %)	1	0.6	1.6
RLQ LAP (n, %)	1	0.6	1.6
Urinary bladder Distention (n, %)	1	0.6	1.6
Cervical LAP (n, %)	1	0.6	1.6
Distented Gall bladder, Gall Sludge (n, %)	1	0.6	1.6
Ileus (n, %)	1	0.6	1.6
Ovarian Cyst (n, %)	1	0.6	1.6
Collection posterior to urinary bladder (n, %)	1	0.6	1.6
Fatty liver (n, %)	1	0.6	1.6
Liver Cyst (n, %)	1	0.6	1.6
Urinary bladder Debris (n, %)	1	0.6	1.6
Intestinal Infection (n, %)	1	0.6	1.6
Kidney Stasis (n, %)	1	0.6	1.6

Further analysis was conducted to explore the clinical significance of the findings. Gender analysis indicated no significant differences in the prevalence of major sonographic findings between males and females. When stratified by age, younger children (aged 5–10 years) showed a lower prevalence of abdominal free fluid compared to older children (aged 11–18 years) (*p* = 0.021). Appendicitis was more frequently observed in patients presenting with RLQ pain (*p* = 0.018) and in those with elevated CRP levels (CRP > 20 mg/L) (*p* = 0.012).

Additionally, renal stones were more commonly found in patients with positive urine protein (*p* = 0.01) and positive urine blood (*p* = 0.001). However, no significant associations were found between clinical features and other major sonographic findings, including intestinal obstruction or mesenteric lymphadenitis. Symptom patterns such as nausea, vomiting, or fever did not show significant correlations with specific sonographic findings. These results suggest that while age, pain location (RLQ), high CRP levels, and positive urine protein or urine blood may influence the prevalence of certain sonographic features, other factors like gender and clinical symptoms may not significantly affect the occurrence of major sonographic findings in pediatric patients with acute abdominal pain (Table 4).

**Table 4:** Association of clinical and laboratory parameters with sonographic findings in pediatric patients

		Yes	No	*P*-value
Abdominal Free Fluid (n, %)	5–10 years old	10 (6.1%)	155 (93.9)	**0.021**
	11–18 years old	3 (33.3)	6 (66.7)	
Appendicitis (n, %)	CRP > 20	4 (17.4)	19 (82.6)	**0.012**
	CRP < 20	4 (2.6)	147 (97.4)	
	Positive RLQ pain	4 (15.4)	22 (84.6)	**0.018**
	Negative RLQ pain	4 (2.7)	144 (97.3)	
Renal Stone (n, %)	Positive Urine Protein	2 (33.3)	4 (66.7)	**0.01**
	Negative Urine Protein	3 (1.8)	165 (98.2)	
	Positive Urine Blood	3 (23.1)	10 (76.9)	**0.003**
	Negative Urine Blood	2 (1.2)	159 (98.8)	

## Discussions

Acute abdominal pain is a frequent and challenging presentation in pediatric emergency departments due to the broad spectrum of underlying conditions and variability in symptom presentation. This study underscores the utility of AUS in evaluating such cases, providing a detailed analysis of sonographic findings and their clinical correlations in a tertiary care setting in Shiraz, Iran. Notably, our findings reveal that the majority of pediatric patients with acute abdominal pain had normal sonographic results, aligning with previous research that reports a significant proportion of cases being nonspecific or self-limiting. Specifically, 53.73% of AUS examinations in prior studies showed no abnormalities, underscoring the importance of clinical judgment in discerning the need for additional imaging or interventions [14]. The study found that active observation was a safe approach for patients without the classical features of appendicitis or peritonitis [15].

Among the abnormal findings, abdominal free fluid was the most prevalent, particularly in older children, followed by conditions such as appendicitis and renal stones. The presence of free fluid is significant as it may indicate an acute inflammatory process, and in pediatric patients, it is commonly associated with conditions such as infection, trauma, or inflammation [16]. Appendicitis, identified in 4.6% of patients in our cohort, emerged as the most critical condition due to its surgical implications. Sonographically, appendicitis presents as an enlarged, non-compressible appendix with increased echogenicity in the surrounding tissue [17]. In Line with this, prior studies have shown that acute appendicitis is the leading cause of abdominal pain in children older than one year, accounting for 68.7% of cases [18]. Supporting this, prior research highlights ultrasound’s sensitivity in diagnosing appendicitis, ranging from 88.5 to 100%, depending on the pathology [19]. Moreover, elevated inflammatory markers, such as CRP, were significantly associated with appendicitis, reaffirming their role in guiding clinical decisions [20].

Although less frequent in this study, intussusception was a notable condition, exhibiting the classic “target sign” on transverse sonographic views. Previous studies have demonstrated 100% sensitivity of ultrasound in diagnosing intussusception [21, 22]. Additionally, the identification of rare conditions such as mesenteric inflammation, vesicoureteral reflux, and ovarian cysts further emphasizes the essential role of AUS in detecting less common but clinically significant pathologies. For example, ovarian pathology and inflammatory bowel diseases become increasingly relevant in older children presenting with acute abdominal pain [23]. Other findings in prior studies included mesenteric lymphadenopathy, splenomegaly, and ascites, which were observed in 7.8%, 3.9%, and 6.7% of patients, respectively [24].

RLQ pain was strongly associated with appendicitis in our study, consistent with its role as a key clinical predictor. Migratory pain and RLQ tenderness have been validated as diagnostic indicators of appendicitis, with the Pediatric Appendicitis Score and Alvarado Score showing increased likelihood ratios (LR, 3.0–3.4) for migratory pain, nausea, and rebound tenderness in identifying appendicitis [25, 26]. Prior research also identified the most common symptoms associated with appendicitis, including fever (64%), emesis (42.4%), decreased appetite (36.5%), cough (35.6%), headache (29.5%), and sore throat (27.0%) [27]. However, symptoms such as nausea, vomiting, and fever alone did not reliably correlate with specific sonographic findings, further emphasizing the indispensable role of AUS in providing definitive diagnoses and guiding clinical decision-making. Recent studies have highlighted the potential diagnostic role of hyperbilirubinemia in pediatric appendicitis, particularly in predicting appendiceal perforation. Azizoğlu et al. reported that elevated bilirubin levels may serve as a marker for complicated appendicitis in children [28]. However, in our study, total and direct bilirubin levels were not routinely measured for the majority of patients presenting with acute abdominal pain. As a result, we were unable to assess the relationship between hyperbilirubinemia and appendicitis outcomes.

Our results align with global trends that emphasize the non-invasive and radiation-free nature of ultrasound, making it particularly suitable for pediatric cases. This modality has significantly influenced surgical decision-making, reducing unnecessary interventions from 27.98 to 7.46% through accurate diagnoses [14]. While ultrasound is highly effective, complementary imaging modalities, such as CT, may be required in select cases to confirm the diagnosis, especially in older children where appendicitis is more prevalent [29]. The study also draws attention to regional variations in the prevalence of sonographic findings, influenced by healthcare infrastructure, referral patterns, and population demographics. These factors underscore the importance of localized studies in tailoring diagnostic protocols. For example, abdominal pain not otherwise specified and acute gastroenteritis remain the most common discharge diagnoses in similar cohorts, with appendicitis diagnosed in 10.2% of pediatric emergency department presentations [20, 30].

Despite the robust findings, the study’s retrospective design introduces potential limitations, including selection bias and the exclusion of patients with inconclusive ultrasound findings. Additionally, the lack of follow-up data prevents evaluation of long-term outcomes of the identified conditions. Future prospective studies with larger sample sizes and longitudinal follow-up are recommended to validate these findings and further explore the clinical impact of sonographic findings on pediatric outcomes.

## Conclusions

In conclusion, this study highlights the significant role of ultrasound in diagnosing acute abdominal pain in pediatric patients at a tertiary care center in Shiraz, Iran. Ultrasound proved effective in identifying both common and rare conditions, with the majority of cases showing normal findings, underscoring the importance of clinical assessment in determining the need for further imaging. Key clinical features such as right lower quadrant pain, elevated CRP levels, and abnormal urine results were linked to specific ultrasound abnormalities, while common symptoms like nausea and vomiting did not reliably correlate with sonographic findings. The study also emphasizes the need for localized research to tailor diagnostic protocols according to regional healthcare settings.

## Data Availability

The datasets used and/or analyzed during the current study are available from the corresponding author on reasonable request.
